# Polymorphisms of *IKBKE* gene are associated with major depressive disorder and panic disorder

**DOI:** 10.1002/brb3.314

**Published:** 2015-02-13

**Authors:** Tanel Traks, Kati Koido, Roman Balõtšev, Triin Eller, Sulev Kõks, Eduard Maron, Innar Tõru, Jakov Shlik, Eero Vasar, Veiko Vasar

**Affiliations:** 1 Department of Physiology, University of TartuTartu, Estonia; 2 Centre of Excellence for Translational Medicine, University of TartuTartu, Estonia; 3 Department of Dermatology and Venerology, University of TartuTartu, Estonia; 4 Department of Psychiatry, University of TartuTartu, Estonia; 5 Department of Pathophysiology, University of TartuTartu, Estonia; 6 Department of Neuropsychopharmacology and Molecular Imaging, Imperial College LondonLondon, U.K; 7 Department of Psychiatry, University of OttawaOttawa, Ontario, Canada

**Keywords:** Genetic association study, *IKBKE*, major depressive disorder, panic disorder, SNP

## Abstract

**Background:**

The immune system has been increasingly implicated in the development of mood and anxiety disorders. Inhibitor of kappa light polypeptide gene enhancer in B cells, kinase epsilon (*IKBKE*) gene encodes IKK*ε* protein that is involved in innate immunity, predominantly antiviral response generation. It also bears pro-inflammatory properties that could affect psychiatric outcomes. In order to investigate the possible role of *IKBKE* gene in major depressive disorder (MDD) and panic disorder (PD), we conducted a case–control genetic association study concerning these disorders.

**Methods:**

In all, 14 SNPs of *IKBKE* gene were genotyped in groups of 391 patients with MDD and 190 patients with PD together with respective 389 and 371 healthy control individuals. The given groups were further divided by gender for additional analyses.

**Results:**

Substantial genetic associations were revealed between *IKBKE* SNPs and MDD (multiple testing adjusted *P* < 0.05) and suggestive associations in case of PD (*P*_adj_ > 0.05). In addition, two SNPs that were only associated with PD among males, also displayed significantly different allele frequencies compared to PD females. This may indicate a specific role of these SNPs in male PD, but caution should be applied here due to the small size of the studied PD males group.

**Conclusions:**

The results of this study confirm our initial findings and indicate a possible role of *IKBKE* gene in mood and anxiety disorders.

## Introduction

Major depressive disorder (MDD) and panic disorder (PD) are both common psychiatric disorders with a high lifetime prevalence that is generally in range 10–15% (Andrade et al. 2003; Kessler et al. 2005) and 3.7–5.1% (Grant et al. 2006; Kessler et al. 2006), respectively, and display a substantial female preponderance. Heritability estimates of 37–38% for MDD (Sullivan et al. 2000; Kendler et al. 2006) and around 40% for PD (Schumacher et al. 2011) suggest a significant role for genetic factors in the development of these diseases. Moreover, due to frequent coexistence of MDD and PD (Johnson and Lydiard 1998), shared genetic risk factors can be expected (Hettema et al. 2006; Mosing et al. 2009).

The classical paradigm relates MDD to impaired monoamine functioning in the central nervous system (CNS). However, the potential contribution of the inflammatory processes of innate immune system to depression is continuously gaining more attention (Loftis et al. 2010; Haroon et al. 2012). According to this concept, the altered expression of various inflammatory mediators, for example, interleukin (IL)-1, IL-6, and tumor necrosis factor (TNF)-alpha lead to changes in multiple aspects of brain functioning and behavior, and are themselves subjected to defective regulation by the hypothalamic-pituitary-adrenal (HPA) axis that begins in the CNS (Capuron and Miller 2011). To date, the genetic studies focusing on candidate genes have predominantly implicated those that are related to monoamine system and are expressed in the CNS as important contributors to MDD, including the serotonin transporter (*SLC6A4*), brain-derived neurotrophic factor (*BDNF*), and tryptophan hydroxylase (*TPH2*) genes (Lohoff 2010). Still, polymorphisms of *IL1B*, *TNF*, C-reactive protein (*CRP*), and other genes related to immune functions have repeatedly been associated with MDD (Bufalino et al. 2013). It is also noteworthy, that although only one among several genome-wide association studies (GWAS) conducted to date has yielded a locus of genome-wide significance (Kohli et al. 2011), a study aiming to replicate previous candidate gene findings by using GWAS data has provided confirmation to four genes as possibly involved in MDD, three among which are related to immune functions (Bosker et al. 2011). The pathology of PD has been related to abnormally sensitive fear network in the CNS (Gorman et al. 2000) and alterations in the activity of the amygdala, the prefrontal cortex and the hippocampus as well as various neurotransmitter and neuroendocrine systems. The evidence for immune alterations has been inconsistent, but shifts in lymphocyte counts and cytokine levels were demonstrated (Park et al. 2005; Hoge et al. 2009). The majority of genetic studies have focused on classic candidate genes related to serotonin, cholecystokinin, dopamine, or adenosine systems, and more recently, the genes involved in immune functions have been added to this group of targets (Maron et al. 2010).

Our previous study explored the single-nucleotide polymorphisms (SNPs) spanning the *IL10* cytokine gene cluster on chromosome 1q32 and revealed a single SNP rs1539243 of the adjacent inhibitor of kappa light polypeptide gene enhancer in B cells, kinase epsilon (*IKBKE*) gene to be associated with the development of MDD and PD, although the result concerning MDD did not remain statistically significant after correction for multiple testing (Koido et al. 2010). This gene encodes inhibitor of *κ*B kinase *ε* protein (IKK*ε*) that is involved in innate antiviral response (Sharma et al. 2003) and has also been identified as oncogenic protein with elevated expression levels in various types of cancers, including glioma (Boehm et al. 2007; Guo et al. 2009; Seo et al. 2009; Guan et al. 2011). In addition to phosphorylating interferon regulatory factor 3 (IRF3) and IRF7 that induce antiviral type I interferon (IFN) expression (Taniguchi and Takaoka 2002; Sharma et al. 2003), IKK*ε* also activates transcription factor NF-*κ*B through different proposed mechanisms which among other effects can cause the production of inflammatory cytokines (Shimada et al. 1999; Peters and Maniatis 2001; Adli and Baldwin 2006; Harris et al. 2006; Mattioli et al. 2006; Sankar et al. 2006). Interestingly, the genetic studies have associated *IKBKE* gene with inflammatory disorders, namely rheumatoid arthritis (RA) and systemic lupus erythematosus (Dieguez-Gonzalez et al. 2009; Sandling et al. 2011). Considering these previous findings and our own preliminary results, we selected additional SNPs of *IKBKE* gene and conducted a more thorough genetic association analysis to detect further associations with MDD and PD.

## Materials and Methods

### Study sample

The participants of this study were recruited from Estonian population: the patients from consecutive outpatients and inpatients at the Clinic of Psychiatry of the Tartu University Hospital and healthy control subjects by a newspaper advertisement in Tartu, Estonia. The MDD (*n* = 391; mean age ±SD 37.1 ± 13.7 years; 114 male/277 female) and PD (*n* = 190; mean age ±SD 38.0 ± 12.8 years; 44 male/146 female) diagnoses of unrelated patients were substantiated by a psychiatric interview and verified by the Mini International Neuropsychiatric Interview (MINI 5.0.0) based on DSM-IV criteria (Sheehan et al. 1998). Controls (389 for MDD; mean age ±SD 37.5 ± 13.5 years; 129 male/260 female, and 371 for PD; mean age ±SD 37.6 ± 13.4 years; 111 male/260 female) were evaluated by the MINI and additional family history interview to exclude those with psychiatric morbidity and a history of major psychiatric disorders in first-degree relatives. All participants were of white European ancestry and living in Estonia. The patients were divided into MDD and PD subgroups according to the primary diagnosis. MDD or PD was considered primary if it was the principal diagnosis at the time of the investigation and/or had an earlier onset in the course of illness. Patients in MDD group were either pure MDD or MDD comorbid with anxiety disorders. Patients in PD group were either pure PD or PD comorbid with mood or other anxiety disorders. The control group was the same for MDD and PD with an exception of reducing the number of male subjects in control group for PD to reflect the respective male/female ratio in PD group. This matching was carried out to address the possible confounding effect of sex, as it has been shown that different genetic factors could have a gender-specific impact on the development of MDD or PD (Kendler et al. 2001; Schumacher et al. 2011). Ultimately, there were no significant differences in age or sex between the patients and healthy controls. In order to conduct an analysis by gender, the MDD and PD groups with their respective controls were divided to male and female subgroups. The resulting groups consisted of 114 MDD males and 277 MDD females with 129/260 controls and 44 PD males and 146 PD females with 111/260 controls. The Ethics Review Committee on Human Research of the University of Tartu approved the study protocol. Each subject provided a written informed consent prior to participation.

### SNP selection and genotyping

Our previous study revealed a single SNP rs1539243 of the *IKBKE* gene that is positioned close to the *IL10* cytokine gene cluster to be associated with PD, and a suggestive result was obtained with MDD (Koido et al. 2010). To further elaborate this result, 13 additional SNPs of *IKBKE* were selected for genotyping and rs1539243 was also re-genotyped. The Tagger tool of Haploview v4.2 program was used to determine tagSNPs of *IKBKE* gene region (de Bakker et al. 2005; Barrett et al. 2005), while even spacing and possible effects on transcription factor binding sites according to Genomatix ElDorado database were considered as additional selection criteria. Genomic DNA was extracted from the 9 mL blood samples and Applied Biosystems SNPlex™ method was used for genotyping (Tobler et al. 2005).

### Data analysis

The Haploview v4.2 program was used for allelic association, linkage disequilibrium (LD), and haplotype analyses between groups of patients and controls, and Hardy–Weinberg equilibrium (HWE) calculations in control group. The Confidence Intervals algorithm was applied to define the haplotype blocks and the resulting blocks were used in the haplotype association test. Comparison of allele or haplotype frequencies between cases and controls was done by chi-squared test. The statistical significance threshold was set to 0.05 for all tests. Ten thousand permutations were used to correct *P* values for errors of multiple testing.

## Results

In all, 14 SNPs of *IKBKE* gene were genotyped in groups of 391 MDD and 190 PD patients and respective 389/371 control individuals. The allele frequencies of all SNPs were similar to those reported within the HapMap Project data for individuals with ancestry from Northern and Western Europe (CEU cohort).

### Allelic association analysis

The results of allelic association analysis for MDD and PD are presented in Table 1. SNP allele frequencies in MDD group compared to control group demonstrated statistically significant differences for two SNPs. Namely, rs2274902 and rs1930437 (*P* values, odds ratios (OR), and 95% confidence intervals (CI) were 0.0013, 0.72 (0.59–0.88) and 0.0007, 1.41 (1.16–1.73), respectively) and these results withstood the correction for multiple testing that involved ten thousand permutations (respective *P* values 0.0291 and 0.0160). Three SNPs showed statistically significant associations with PD – rs1539243, rs1953090, rs11117909, but the respective *P* values of 0.0112 (OR 1.50 (CI: 1.09–2.05)), 0.0339 (OR 0.73 (CI: 0.55–0.98)), and 0.0256 (OR 1.49 (CI: 1.05–2.13)) did not survive the corrections for multiple testing. Rs1930438 showed a trend toward association with PD, but narrowly missed the significance threshold.

**Table 1:** Results of allelic association analysis

#	SNP	Location	Major/minor alleles	MAF (%)		Chi square	MDD *P* value	MAF (%)		Chi square	PD *P* value
				MDD cases	MDD controls			PD cases	PD controls		
1	rs1930438	5′ near gene	G/A	16.7	15.7	0.305	0.581	20.6	15.9	3.805	0.051
2	rs17020312	5′ UTR	A/C	4.3	4.0	0.129	0.720	4.2	3.9	0.06	0.807
3	rs2274902	Intron 2	G/A	41.6	49.7	10.33	0.001*	47.4	49.2	0.333	0.564
4	rs1930437	Intron 2	T/G	55.8	47.1	11.574	<0.001*	49.5	48.0	0.227	0.633
5	rs1539243	Exon 4	C/T	17.6	16.0	0.7	0.403	22.0	15.8	6.436	**0.011**
6	rs1953090	Intron 4	A/C	30.8	28.5	0.996	0.318	23.0	29.0	4.501	**0.034**
7	rs11117909	Intron 6	G/A	12.7	12.2	0.084	0.772	16.3	11.5	4.981	**0.026**
8	rs2297543	Intron 8	G/A	22.4	20.4	0.901	0.343	18.3	21.0	1.177	0.278
9	rs17021877	Exon 10	G/A	0.8	1.0	0.321	0.571	0	1.0	3.638	0.057
10	rs11118092	Intron 13	C/T	35.8	38.0	0.778	0.378	36.5	37.4	0.091	0.763
11	rs11118132	Intron 15	G/C	33.7	32.3	0.355	0.552	33.3	32.6	0.067	0.797
12	rs2336940	Intron 18	G/C	15.9	14.8	0.355	0.551	17.2	15.0	0.874	0.350
13	rs3748022	Exon 22	C/T	17.6	17.8	0.019	0.891	16.1	18.5	1.054	0.305
14	rs15672	3′ UTR	G/A	48.0	50.0	0.647	0.421	45.8	49.3	1.263	0.261

MDD, major depressive disorder; PD, panic disorder; MAF, minor allele frequency.

*P* values ≤0.05 are highlighted in bold.

* Designates *P* values ≤0.05 after 10,000 permutations.

Additional analysis by gender resulted in no significant associations in male MDD group and two associations in female MDD group that concerned the same SNPs as in the whole MDD group – rs2274902 and rs1930437. The respective *P* values of 0.0044 (OR 0.70 (CI: 0.55–0.87)) and 0.0037 (OR 1.43 (CI: 1.12–1.82)) did not remain significant after permutations. In male PD group, two significant results were evident, involving rs1953090 and rs2297543 with respective *P* values of 0.0013 (OR 0.34 (CI: 0.18–0.67)) and 0.0456 (OR 0.49 (CI: 0.24–1)). Association with the former SNP was present in whole PD group and remained significant after permutations (*P* = 0.0242). The female PD group produced one association for rs11117909 (*P* = 0.0485, OR 1.51 (CI: 1–2.28)) that was associated in whole PD group, and the other for rs1930438 (*P* = 0.0449, OR 1.45 (CI: 1.01–2.1)). These did not remain significant after permutations. Additionally, rs1539243, that was associated in whole PD group, showed a trend toward association (*P* = 0.0532).

### Haplotype analysis

**Figure 1:**
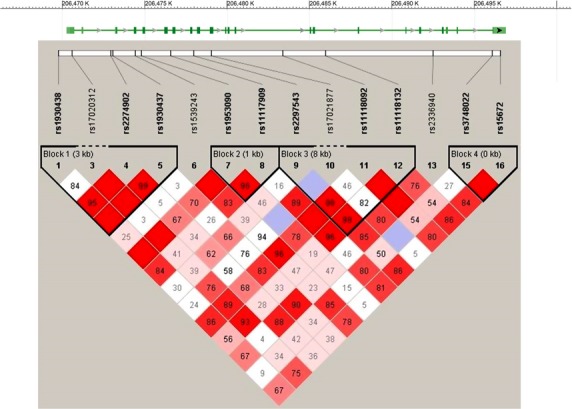
Genotyped SNPs of *IKBKE* gene region. The image of LD pattern was generated using the Haploview v4.2 program and black boxes indicate haplotype blocks in MDD association analysis.

Four haplotype blocks were formed by 10 of the genotyped SNPs (Fig.1). Two haplotypes of Block 1 (consisting of rs1930438, rs2274902, and rs1930437) were associated with MDD (Table 2). Specifically, haplotypes GAT and GGG with respective *P* values of 0.0015 (OR 0.72 (CI: 0.59–0.88)) and 0.0022 (OR 1.38 (CI: 1.12–1.70)) that remained below the significance threshold after corrections for multiple testing (permutation *P* values 0.0332 and 0.0479, respectively). While the frequency of the GAT haplotype was lower among MDD patients (41.3%) compared to control individuals (49.3%) thus indicating a possible protective effect, the frequencies of GGG haplotype were reversed (38.9% in MDD group and 31.5% in control group), thereby suggesting a possible promoting effect, that is, risk haplotype. Two haplotypes of Block 2 consisting of rs1539243 and rs1953090 were associated with PD (Table 3). Haplotype CC was protective (22.7% in PD group and 28.9% in control group, *P* = 0.0272, OR 0.72 (CI: 0.54–0.97)), whereas TA was a risk haplotype (21.6% in PD group and 15.8% in control group, *P* = 0.0154, OR 1.47 (CI: 1.08–2.02)), although these results did not survive the correction for multiple testing. Alternatively, in MDD analysis this block was formed by rs1953090 and rs11117909 (Fig.1). Haplotype CG of Block 4 consisting of rs3748022 and rs15672 also produced a suggestive result as a risk haplotype (38.1% in PD group and 32.1% in control group, *P* = 0.0441, OR 1.30 (CI: 1.01–1.69)), but again, this did not survive the correction for multiple testing (Table 3).

**Table 2:** Results of Block 1 haplotype analysis in MDD group

rs1930438	rs2274902	rs1930437	Haplotype frequencies (%)			Chi square	*P* value
			Total	Cases	Controls		
G	A	T	45.3	41.3	49.3	10.046	0.002*
G	G	G	35.3	38.9	31.5	9.348	0.002*
A	G	G	16.0	16.5	15.5	0.302	0.583
G	G	T	3.3	3.0	3.5	0.374	0.541

MDD, major depressive disorder.

*P* values ≤0.05 are highlighted in bold.

* Designates *P* values ≤0.05 after 10,000 permutations.

**Table 3:** Results of Block 2 and Block 4 haplotype analysis in PD group

Block 2		Block 4		Haplotype Frequencies (%)			Chi square	*P* value
rs1539243	rs1953090	rs3748022	rs15672	Total	Cases	Controls		
C	A			55.3	55.4	55.3	0.0	0.986
C	C			26.8	22.7	28.9	4.876	**0.027**
T	A			17.8	21.6	15.8	5.875	**0.015**
		C	A	48.2	45.8	49.3	1.239	0.266
		C	G	34.1	38.1	32.1	4.051	**0.044**
		T	G	17.7	16.1	18.6	1.088	0.297

PD, panic disorder.

*P* values ≤0.05 are highlighted in bold.

When analyzing by gender, no associations were present in male MDD group and two associations that coincided with the ones in whole MDD group were present in female MDD group. Namely, these concerned the haploptypes GAT and GGG of Block 1 with respective *P* values of 0.0085 (OR 0.72 (CI: 0.57–0.92)) and 0.0113 (OR 1.39 (CI: 1.08–1.78)), and did not remain significant after permutations. In male and female PD groups rs1539243 and rs1953090 did not form a haplotype block. Therefore, the Block 2 associations present in whole PD group cannot be compared with the results from these subgroups. One haplotype association was evident in male PD group (*P* = 0.0389, OR 2.04 (CI: 1.03–4.05)). It involved the haplotype GCC of Block 3 that is formed by rs2297543, rs11118092, and rs11118132 and did not remain significant after permutations. No further associations were detected in male or female PD groups.

## Discussion

The findings of our previous study suggested a possible role for *IKBKE* gene in PD and MDD (Koido et al. 2010). Thus, additional SNPs of *IKBKE* gene were genotyped, resulting in the detection of several allelic and haplotype associations in both MDD and PD study groups. Two single marker and two haplotype associations were identified for MDD and these affirmingly survived the correction for multiple testing. Three single marker and three haplotype associations were identified for PD, although these did not remain significant when the correction for multiple testing was applied. However, it should be noted, that there were roughly half as many PD patients in the analysis as MDD patients and given a larger sample size, the similar allele and haplotype frequencies could result in more significant results. The formerly associated SNP rs1539243 (Koido et al. 2010) produced a weaker association for PD in current analysis and did not meet the significance threshold in case of MDD. This contrast can be reasoned by the recognition that the present control and patient groups were not identical to those that were assembled previously. While the majority of samples were unchanged, a significant portion of controls and patients were omitted and replaced by new samples. When analyzing by gender, a number of differences in allelic and haplotype associations occurred. Despite this, in MDD males and females, the associated allele and haplotype frequencies were actually similar, and the observed discrepancies could be attributed to differences in sample sizes. The same can be argued for PD subgroups with the exception of SNP rs2297543 (G allele case/control frequencies (%) in PD males vs. PD females: 87.2/77 vs. 80.1/79.8) together with Block 3 haplotype GCC (19.1/10.4 vs. 13.5/12.5), and most notably rs1953090 (A allele: 86.4/68.5 vs. 74.1/72.1). These displayed significant differences only in PD males compared to their controls. This may indicate that these polymorphisms specifically influence PD susceptibility in males, but insists caution when considering the small sizes of PD males and their control groups.

The *IKBKE* gene is composed of 22 exons and is located in chromosomal region 1q32. According to available literature, the genetic polymorphisms of this region have been associated with both MDD and PD. Candidate gene studies have implied arginine vasopressin receptor 1B (*AVPR1B*) and plexin A2 (*PLXNA2*) genes as possible contributors to these diseases (van West et al. 2004; Wray et al. 2007; Keck et al. 2008) and a recent GWAS proposed plakophilin 1 (*PKP1*) gene for this role in PD, although the result was not subsequently replicated in an independent sample (Otowa et al. 2009, 2010). Genetic linkage studies have resulted in modest evidence for 1q involvement in MDD and PD (Gelernter et al. 2001; Zubenko et al. 2003) and more specifically 1q32 in PD (Smoller et al. 2001). Additionally, this region has emerged as one of the susceptibility loci for other psychiatric disorders such as bipolar disorder and schizophrenia (Detera-Wadleigh et al. 1999; Gurling et al. 2001). Taken together, these findings highlight 1q32 as one of the potential susceptibility loci for MDD and PD, and the *IKBKE* variants within it could represent some of the truly causal factors.

IKK*ε* kinase is predominantly expressed in immune cells and can be activated by double-stranded (ds) DNA, dsRNA, and viral infection (Shimada et al. 1999; Fitzgerald et al. 2003; Hemmi et al. 2004; Ishii et al. 2006) as well as inflammation-inducing lipopolysaccharide (LPS) and cytokines (Shimada et al. 1999; Kravchenko et al. 2003; Hemmi et al. 2004). On the one hand, this leads to subsequent activation of IRF3 and IRF7 that induce type I IFN expression which is associated with anti-viral response (Taniguchi and Takaoka 2002; Sharma et al. 2003), but has also been implicated in sustaining chronic inflammation (Lee et al. 2009). The other notable effect is the activation of NF-*κ*B, a key regulator of immune responses to infection and inflammatory processes, and this can elevate the production of inflammatory cytokines (Shimada et al. 1999; Sankar et al. 2006). Of note, NF-*κ*B itself has in turn been identified as an activator of IKK*ε* (Wang et al. 2005). Considering these relations between IKK*ε* and inflammatory mediators and the role of inflammatory processes in mood and anxiety disorders, it can thus be proposed that in the event of altered IKK*ε* mRNA expression or protein functioning it may contribute to impaired immune regulation and therefore to the development of these psychiatric conditions. For instance, it has been demonstrated that NF-*κ*B signaling is a key to developing depressive-like behaviors under chronic stress (Koo et al. 2010), and hence there is a possibility of modulation by IKK*ε*. Another supportive finding to this notion is the detection of IKK*ε* in fibroblast-like synoviocytes of RA and osteoarthritis patients that suggests its possible involvement in chronic inflammatory conditions (Sweeney et al. 2005). Interestingly, the intronic SNP rs1930437 that was associated with MDD in this study affects the binding site of c-Rel, a transactivating component of a dimeric NF-*κ*B, as evident from JASPAR transcription factor database search by ConSite web-based tool (Sandelin et al. 2004). Namely, the substitution of allele G for allele T disrupts the c-Rel binding sequence and could possibly abolish the enhancer function of NF-*κ*B on IKK*ε* mRNA transcription. The G allele was significantly more frequent among MDD patients compared to control subjects (55.8 vs. 47.1%) and this may indicate a more active *IKBKE* transcription that promotes inflammatory mediators and eventually influences psychiatric outcomes. The remaining four associated SNPs similarly do not cause changes in the peptide sequence of IKK*ε* protein. It is possible that these SNPs are in LD with other polymorphisms that affect *IKBKE* transcription (for rs2274902 this could be the above described rs1930437) or with nonsynonymous mutations that may exist in the studied population as rare variants. For example, the three SNPs associated with PD are located in the region of exons that encode the kinase domain of IKK*ε* (Shimada et al. 1999) and polymorphisms modifying its structure might be reflected in the observed genetic associations.

The presented findings should be interpreted with caution due to methodological limitations. The sample sizes were small, especially the PD patients group. Both MDD and PD groups contained subjects with comorbid diagnoses. Different SNPs were associated with MDD compared to PD and one possibility to interpret this is that distinct genetic or functional changes in *IKBKE* can influence the development of MDD and PD. However, this could also indicate false positive results, especially in case of PD, as they failed the correction for multiple testing. Finally, the majority of the sample and one genotyped SNP overlapped with our previous report (Koido et al. 2010), and thus it cannot be considered as an independent replication study, hindering the significance of current findings.

## Conclusion

Our study contributes to general research that explores the role of innate immune inflammatory processes in MDD and PD. The presented results confirm our initial findings and indicate a possible role of *IKBKE* gene in mood and anxiety disorders. Several single marker and haplotype associations were evident for both MDD and PD, although the ones concerning MDD were more convincing. However, the noticeable limitations of this investigation should be considered when interpreting this data. Therefore, replication genetic studies in different samples along with functional analyses are required to confirm the relevance of current findings. By identifying the various genetic factors and achieving a more thorough understanding of the immune system in MDD and PD, this could ultimately lead to advancements in prevention and treatment.
